# Fabrication of Graphene Aerogels with Heavily Loaded Metallic Nanoparticles

**DOI:** 10.3390/mi8020047

**Published:** 2017-02-07

**Authors:** Chen Shen, Elizabeth Barrios, Matthew McInnis, Joseph Zuyus, Lei Zhai

**Affiliations:** 1 NanoScience Technology Center, University of Central Florida, Orlando, FL 32826, USA; nstcshenchen@knights.ucf.edu (C.S.); eab143@Knights.ucf.edu (E.B.); matt.d.mcinnis@gmail.com (M.M.); joezuyus@gmail.com (J.Z.); 2 Department of Materials Science and Engineering, University of Central Florida, Orlando, FL 32826, USA; 3 Department of Chemistry, University of Central Florida, Orlando, FL 32826, USA

**Keywords:** graphene, aerogels, metal nanoparticles, hydrogen sensors

## Abstract

Natural biomaterials with hierarchical structures that enable extraordinary capability of detecting chemicals have inspired the interest in producing materials that can mimic these natural structures. This study reports the fabrication of hierarchically-structured, reduced graphene oxide (rGO) aerogels with heavily loaded palladium (Pd), platinum (Pt), nickel (Ni), and tin (Sn) metallic nanoparticles. Metal salts chelated with ethylenediaminetetraacetic acid (EDTA) were mixed with graphene oxide (GO) and then freeze-dried. The subsequent reduction produces rGO/metal nanoparticle aerogels. SEM and EDS results indicated that a loading of 59, 67, 39, and 46 wt % of Pd, Pt, Ni, and Sn nanoparticles was achieved. Pd/rGO aerogels of different Pd nanoparticle concentrations were exposed to H_2_ gas to monitor the resistance change of the composites. The results suggest that rGO aerogels can achieve a higher nanoparticle loading by using chelation to minimize electrostatic interactions between metal ions and GO. Higher loading of Pd nanoparticles in graphene aerogels lead to improved hydrogen gas sensing performance.

## 1. Introduction

**Figure 1:**
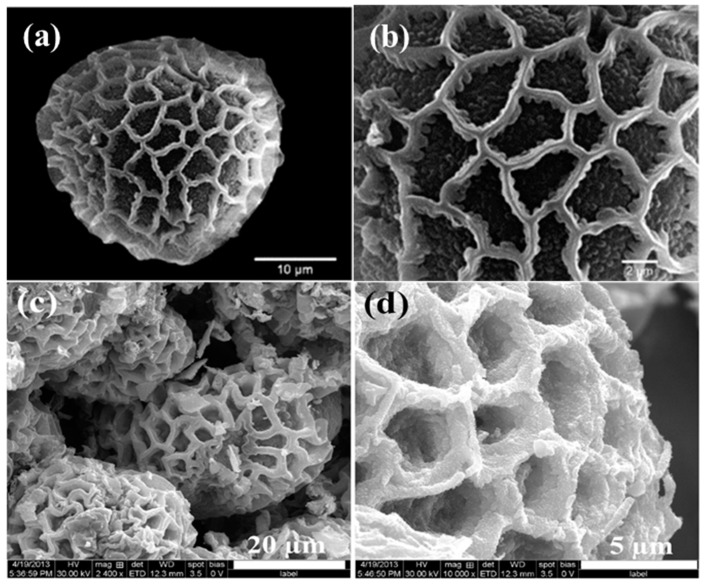
(**a**,**b**) are SEM images of Balsaminaceae pollen with a microscale porous network and nanoscale structures on the surface [2]; (**c**,**d**) are SEM images of constructed SnO_2_ structures for gas sensing created using *Peltophorum pterocarpum* pollen grains as a template [3].

Nature has produced remarkable materials possessing unique characteristics and behaviors that inspire scientists to replicate their structures synthetically [1]. Unlike synthetic materials, many natural biomaterials possess a hierarchical structure on many different size scales that have a complex interaction between each scale, leading to very unique behaviors. For example, the biomolecules in pollens self-assemble to form a connective, highly porous network that enables efficient mass transport [2]. Additionally, nanostructures on pollens possess a very large internal surface area, endowing many possible sites for surface reactions to occur (Figure 1a,b) [3]. In pollen grains, these two features enable a sensitive response of the pollen-stigma recognition, fertilization, and the defense mechanism [4,5]. As such, the hierarchical porous network platform demonstrated in the pollen grain provides the possibility to manufacture a highly sensitive and efficient gas sensor. These possibilities have led to many researchers trying to fabricate biomimetic-sensing systems to advance today’s gas sensing technologies [2,3,6,7,8,9,10]. For example, tin(IV) oxide (SnO_2_) porous particles with large surface area have been produced using pollens as templates (Figure 1c,d) [3]. In this work, we demonstrate a strategy to achieve a similar hierarchical porous network for hydrogen sensing capabilities through the loading of metallic nanoparticles onto a graphene aerogel motif.

Graphene is a planar monolayer of carbon atoms arranged in a honeycomb lattice and has demonstrated very intriguing electrical properties, high strength, and high in-plane electrical conductivity. Additionally, graphene has a very large surface-to-weight ratio, making it highly suitable for sensor applications. For example, various metal nanoparticles have been attached to graphene to detect chemicals such as hydrogen peroxide [11,12,13] and hydrogen [14]. The hierarchical porous structure demonstrated in the pollen grains can be achieved in graphene through the creation of a graphene aerogel (GA) decorated with nanostructures [15] where aerogels provide a network for charge transport and nanostructures grant large surface area to interact with hydrogen gas. GA is a nanoscale graphene network with low density, large open pores, and high specific surface area. With unique electrical properties and a large surface area of graphene, GAs are promising materials for gas sensing and energy storage applications [16,17,18,19,20,21,22,23,24,25,26,27,28,29,30,31]. Historically, GA is produced through freeze-drying a graphene oxide (GO) hydrogel, where the graphene oxide is synthesized via a modified Hummer’s method. The freeze-dried GO aerogel is then reduced to produce a reduced graphene oxide (rGO) aerogel. This method produces physical crosslinks between the graphene sheets that are oriented in the freezing direction, creating long, continuous open pores throughout the GA [31,32,33]. For the use in gas sensing applications, GA is often functionalized with metallic nanoparticles like palladium, platinum, nickel, tin, and so on [34]. 

Loading metallic nanoparticles onto a graphene surface requires mixing GO dispersion with a metal salt solution. The mixture is freeze-dried and then reduced to produce graphene/metallic nanoparticles composites [35,36]. However, the loading capacity of metallic nanoparticles on graphene sheets is limited by the bridging effect between GO and metal cations, and this effect diminishes the gas-sensing performance of the materials [35,37]. To overcome this limitation, chelating agents such as ethylenediaminetetraacetic acid (EDTA) can be used to screen the electrostatic interaction between metal cations and GO, thus increasing the density of metallic nanoparticles exposed on the surface of GO sheets [38].

Herein, we report an effective approach for loading a large amount of metallic nanoparticles onto the surface of graphene aerogels using EDTA. These functionalized GO aerogels were fabricated by creating a metal salt solution containing EDTA prior to the introduction of the aqueous GO dispersion. The resulting GO aerogels loaded with metal ions were reduced to obtain the rGO (i.e., graphene) aerogels with nanoparticles. In this work, we demonstrate the successful preparation of palladium, platinum, nickel, and tin nanoparticle-decorated graphene aerogels and the fabrication of a hydrogen gas sensor using palladium nanoparticle-loaded graphene aerogels.

## 2. Materials and Methods

### 2.1. Materials and Instruments

Sulfuric acid, potassium permanganate, hydrogen peroxide, and 0.5 M ethylenediaminetetraacetic acid (EDTA) solution were purchased from Fisher Scientific. Tin chloride pentahydrate (SnCl_4_∙5H_2_O), nickel chloride hexahydrate (NiCl_2_∙6H_2_O), palladium chloride (PdCl_2_), and chloroplatinic acid (H_2_PtCl_6_) were purchased from Alfa Aesar. Graphite powders (microfyne grade) were purchased from Dixon Inc. All chemicals were used as received without further purification.

A Labconco freeze drying chamber (FreeZone 1) was used for the lyophilization of the graphene oxide hydrogels. The Labcono lyophilizer was operated at a temperature of −52 °C under a pressure of 0.02 mbar. VWR vacuum oven was used for the vacuum drying of GO powders. BRANSON digital horn sonifier was used to disperse GO in water. A Thermo Scientific tube furnace (Lindberg Blue M) was used for the hydrogen reduction of the freeze-dried graphene oxide aerogels. 

### 2.2. Synthesis of Graphene Oxide (GO) Using Modified Hummers’ Method

Graphene Oxide (GO) was prepared by modified Hummers method using graphite powder. Typically, 1 g of graphite powder was added to a 500 mL beaker that was immersed in an ice bath, followed by a slow addition of 50 mL concentrated sulfuric acid and 6 g of potassium permanganate while stirring. The mixture was stirred for an additional hour at 30 °C. 80 mL of deionized water (DI water) was added dropwise to the homogeneous mixture. After the addition of DI water, the beaker was then heated to 60 °C and maintained for 1 h. 6 mL of 30% H_2_O_2_ was then added into the beaker. The heat was turned off after 5 min. After the mixture was cooled to room temperature, the crude products were washed with DI water followed by centrifugation to obtain the product. The washing was finished when the supernatant had a pH above 5. The wet GO was then dried in a vacuum oven at room temperature to avoid thermal reduction during the drying process [39,40].

### 2.3. Preparation of GO Aerogels Loaded with Metal Salts

**Figure 2:**
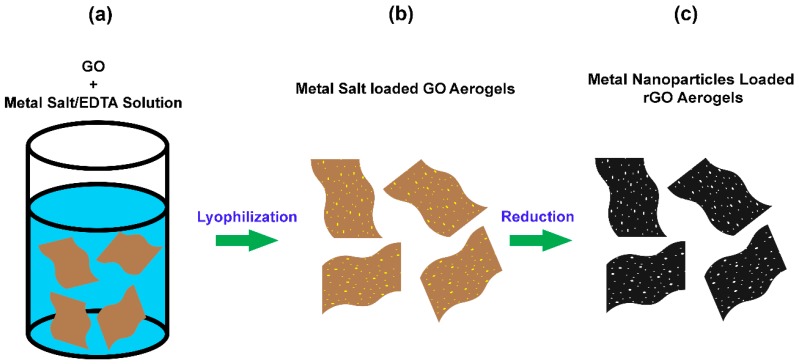
Schematic Illustration of the formation of metal nanoparticles decorated rGO aerogels. (**a**) GO + Metal Salt/EDTA Solution. (**b**) Metal Salt loaded GO Aerogels. (**c**) Mental Nanoparticles Loaded Rgo Aerogels.

In a typical preparation, 1 mM of a metal salt (i.e., Pd, Pt, Ni, or Sn salts) was added to a 10 mL 0.1 M EDTA solution. Thus, the molar ratio between the metal salt and EDTA was 1:1. Most solutions were prepared at room temperature. However, the PdCl_2_/EDTA solution was heated at 90 °C for 15 min to fully dissolve the metal salt, and the solution was cooled to room temperature prior to the next step. An aqueous GO dispersion was prepared by using a horn sonifier. A mixture of 200 mg of GO and 10 mL of DI water was sonicated for 30 min to form an aqueous GO dispersion. As shown is Figure 2a, the completely dissolved metal salt/EDTA solutions were mixed with 20 mg/mL GO dispersion with a volume ratio of 1:1 to form a metal salt/GO dispersion. The dispersion was then transferred to several 2 mL plastic cuvettes after vigorous mixing was applied. Dispersions in cuvettes were frozen using dry ice for the following lyophilization. After an overnight lyophilization, bulk metal salt loaded GO aerogels were formed as shown in Figure 2b.

### 2.4. Preparation of Reduced Graphene Oxide (rGO) Aerogels Decorated with Metal Nanoparticles 

The metal salt loaded GO aerogels shown in Figure 2b went through vapor phase reduction, and an atmosphere of hydrazine vapor mixed in nitrogen was used during this reaction. The reduction was conducted at 90 °C for one hour. Epoxide functional groups on the basal plane of GO were removed by hydrazine vapor to avoid potential structure damage of aerogels in the next hydrogen reduction step [39,41]. Once the aerogels were cooled down from hydrazine reduction, they were further reduced by pure hydrogen gas in a pure hydrogen atmosphere at 400 °C for 1 h and at 900 °C for 10 min. The hydrogen flow rate was kept at 30 SCCM. Such two steps reduction produced rGO aerogels decorated with metal nanoparticles as shown in Figure 2c, and the color of aerogels was converted from brown to black during the reduction process. To form tin decorated rGO aerogels, hydrogen reduction only needs to be conducted at 400 °C for one hour as tin nanoparticles would flow away if higher temperatures were applied.

### 2.5. Characterization

The morphologies of the aerogels were characterized by using a scanning electron microscope operating at 10 KeV (SEM, ZEISS ULTRA 55, Zeiss, Dublin, CA, USA) and was coupled with energy-dispersive X-ray spectroscopy (EDS) to investigate the surface chemistry of the aerogels. The chemical states of elements were analyzed by X-ray photoelectron spectroscopy (XPS, PHI 5400, Ulvac-PHI, Inc., Kanagawa, Japan). The surface area of aerogels was measured by a Nova 4200e Brunauer, Emmett, and Teller (B.E.T.) surface area analyzer (Quantachrome Instruments, Boynton Beach, FL, USA).

### 2.6. Sensing Hydrogen Gas Using rGO Aerogels Decorated with Pd Nanoparticles

In order to determine the influence of processing and experimental conditions of the aerogels on hydrogen sensing, rGO aerogels attached to electrical leads was inserted into a homemade flow chamber. The flow chamber was placed in a water bath with stable temperature, and a thermocouple was added to the interior of the flow chamber to diminish the affect from the exterior temperature. Conductometric response (i.e., resistance change of rGO aerogels) to hydrogen gas was carried out by monitoring the current through each aerogel under the condition of a steady flow of argon gas with periodic inclusion of 1000 ppm hydrogen gas into the stream. The relative change in resistivity [(R_g_ − R_0_)/R_0_%] (R_0_ and R_g_ are the resistance of the device before and after the exposure to hydrogen gas, respectively.) was calculated from the current and the voltage applied. These values were plotted as a function of time after baseline correction.

## 3. Results and Discussion

The material fabrication started with a solution containing equal moles of a metal salt and a ligand, specifically EDTA, in this study. EDTA was crucial in order to successfully form GO aerogels during lyophilization. If a strong ligand like EDTA was not used, the strong electrostatic interactions were between positively charged metal ions and the negatively charged functional groups on the edge of GO, usually the carboxylic acid groups, caused the gelation of GO [42]. Such a GO hydrogel would shrink significantly during the lyophilization. As a result, GO aerogels with porous microstructures were not formed, leading to the loss of the advantages of the aerogels originating from this unique microstructure. On the other hand, if metal salts are mixed with EDTA before being added to the GO suspension, the formation of metal-EDTA complexes eliminated the electrostatic interactions between metal ions and GO [43,44,45]. Therefore, van der Waals interactions between the GO sheets became the major interactions in the mixtures, which is the same as when only GO was dispersed in water. As a result, nearly no shrinkage was observed after the lyophilization, and aerogels containing metal salts were successfully formed. After successfully creating the aerogels containing metal salts, the GO flakes and metal ions in the aerogels were converted to rGO sheets and metal nanoparticles during the high temperature hydrogen reduction.

**Figure 3:**
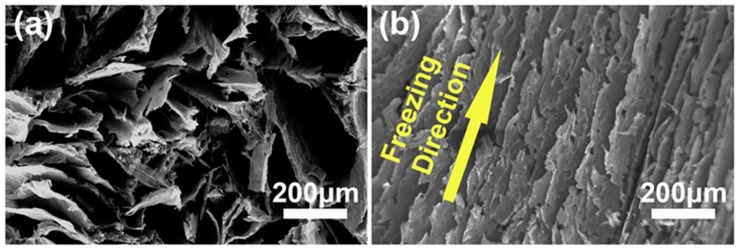
(**a**) SEM image of the cross-sectional area; (**b**) SEM image of the longitudinal section.

The microstructure of a typical rGO aerogel decorated with metal nanoparticles was examined by SEM as shown in Figure 3. The stacking of the rGO sheets can be clearly seen from the cross-sectional image in Figure 3a. This structure indicates that the van der Waals interactions among the rGO sheets remained even after the hydrogen reduction process. Also, it is shown that the pore sizes in the aerogels are in the sub-millimeter range. The interconnected porous graphene framework with a significant directionality in the longitudinal direction was realized (Figure 3b). The well-defined 3D microstructure is due to the directional freezing resulting from placing dry ice beneath the cuvettes filled with metal salt/GO dispersions.

**Figure 4:**
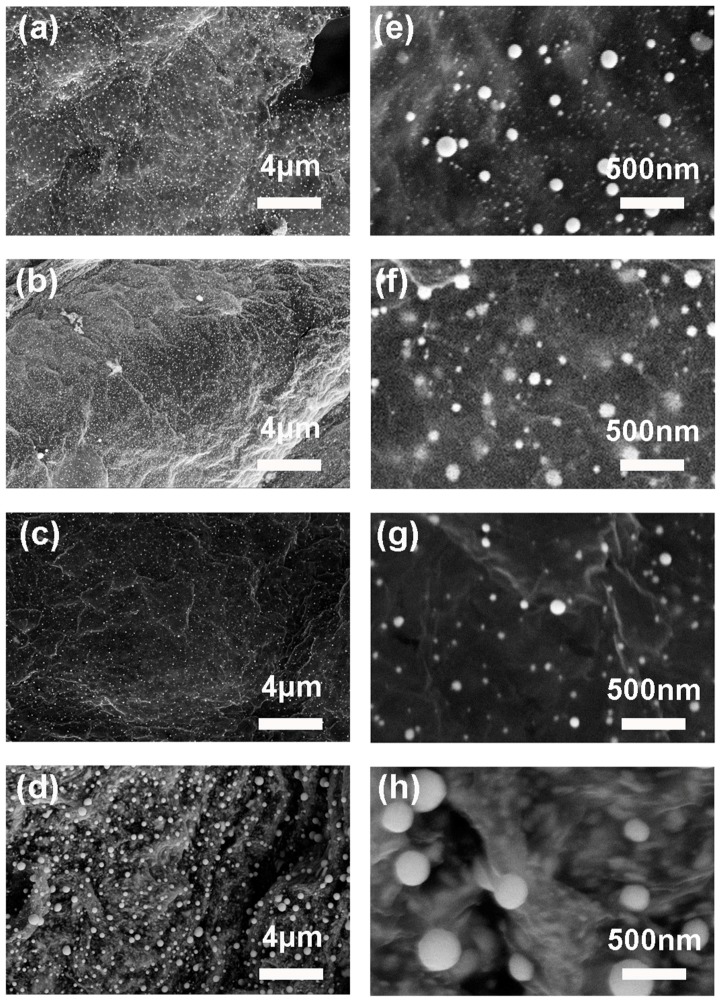
SEM images of the (**a**,**e**) Pd; (**b**,**f**) Pt; (**c**,**g**) Ni; and (**d**,**h**) Sn nanoparticles loaded aerogels formed after hydrogen reduction.

The loading of the metal nanoparticles was verified by the SEM under higher magnifications as shown in Figure 4. The morphologies of the aerogels loaded with palladium, platinum, nickel, and tin nanoparticles are shown in Figure 4a,e; Figure 4b,f; Figure 4c,g; and Figure 4d,h, respectively. The presence of ripples and wrinkles on the sheets verified the existence of rGO sheets. These features are typical in rGO sheets and mainly result from the stress produced at elevated temperatures during hydrogen reduction process. After hydrogen reduction, metal ions chelated with EDTA on the rGO sheets were reduced into their metallic state. The sizes of palladium, platinum, and nickel metal nanoparticles are comparable, which ranged from 10 to 120 nm in diameter. The tin nanoparticles loaded on rGO sheets have larger sizes compared with the other three metal nanoparticles. Particles as large as 400 nm in diameter were observed. However, small tin nanoparticles with diameters around 50 nm can still be observed.

**Figure 5:**
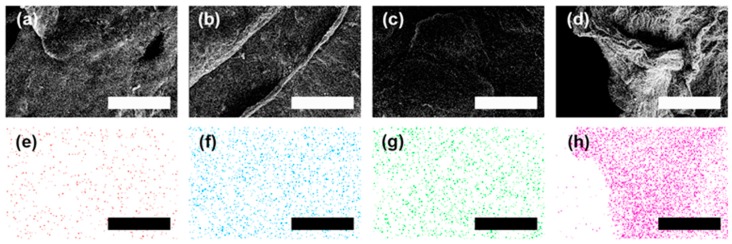
SEM images of (**a**) Pd, (**b**) Pt, (**c**) Ni, and (**d**) Sn loaded rGO aerogels and the corresponding EDS spatial mapping images of loaded (**e**) Pd, (**f**) Pt, (**g**) Ni, and (**h**) Sn on rGO sheets. The scale bar is 20 μm.

To verify the distribution of metal nanoparticles on rGO sheets, the metal loaded aerogels were characterized by using EDS elemental mapping. The SEM images of metal loaded aerogels and the mapping of the loaded metal in the same area are shown Figure 5. The EDS mapping results of rGO aerogels loaded with palladium, platinum, nickel, and tin are shown in Figure 5a,e; Figure 5b,f; Figure 5c,g; and Figure 5d,h, respectively. Figure 5a–d shows SEM images of the area characterized for EDS elemental mapping. Uniform elemental distribution of metals is clearly indicated by Figure 5e–h. Among them, Figure 5e–h presents the elemental mapping of palladium, platinum, nickel, and tin on their corresponding rGO sheets in aerogels, respectively. These results confirm the successful loading of metal particles onto rGO aerogels with excellent uniformity. 

**Figure 6:**
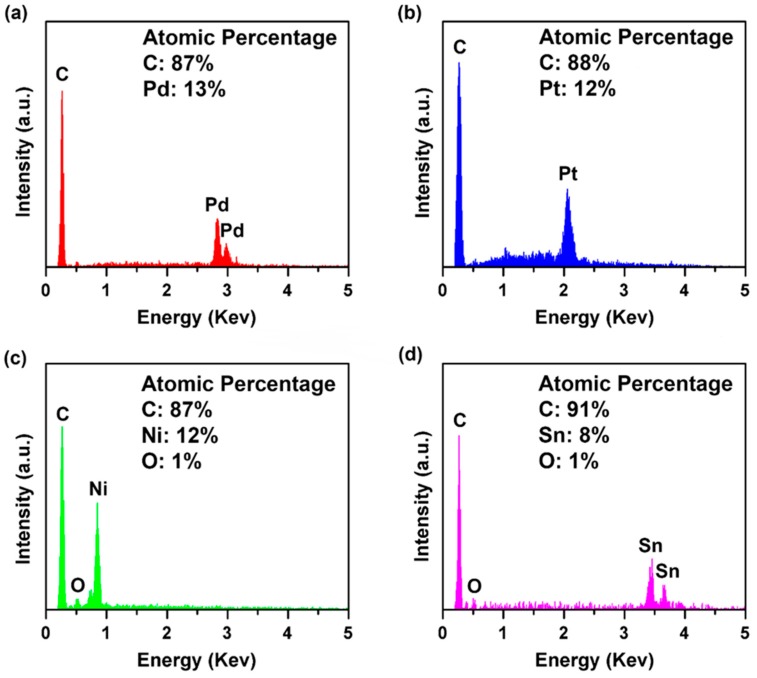
EDS spectra of (**a**) palladium, (**b**) platinum, (**c**) nickel, and (**d**) tin loaded rGO aerogels after hydrogen reduction.

The amount of loaded metal nanoparticles in rGO aerogels after hydrogen reductions was also characterized by EDS. Based on the EDS spectra in Figure 6, carbon and the loaded metals are the predominant components of the aerogels after hydrogen reduction. Oxygen in the palladium and platinum loaded aerogels are not detectable to give a reliable composition with the oxygen; however, small peaks at 0.525 eV can be seen for these two trials. Oxygen in the nickel and tin loaded aerogels were detected in quantifiable quantities and is likely attributed to the oxides that formed on the surfaces of the aerogels, which will be discussed further in the XPS studies. For the aerogels containing tin, the excess oxygen content compared with the other samples is also due to the incomplete reduction of the rGO that occurred during reduction conducted at the significantly lower temperature of 400 °C. The loading efficiency of the metal nanoparticles was evaluated by analyzing the EDS spectra in Figure 6, and the mass percentage of metal nanoparticles in rGO aerogels after hydrogen reduction was calculated to be 59%, 67%, 39%, and 46% for Pd, Pt, Ni, and Sn nanoparticles, respectively. Compared with existing approaches of loading metal nanoparticles to graphene (around 9 at. % and 26 wt %) [46,47], our method grants much higher loading efficiencies.

**Figure 7:**
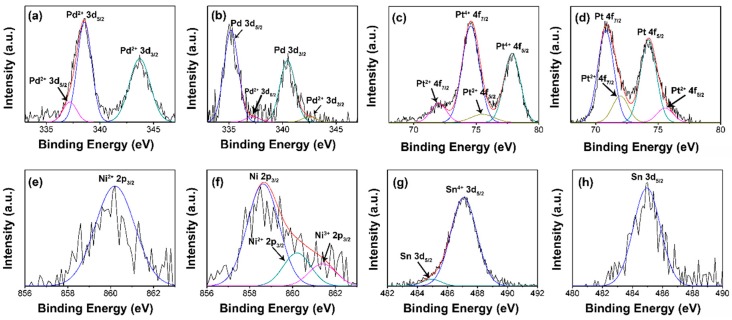
Deconvoluted XPS spectra of the loaded metal in the aerogels. Pd in Pd loaded aerogels (**a**) as prepared and (**b**) after reduction, Pt in Pt loaded aerogels (**c**) as prepared and (**d**) after reduction, Ni in Ni loaded aerogels (**e**) as prepared and (**f**) after reduction, Sn in Sn loaded aerogels (**g**) as prepared and (**h**) after reduction are shown.

**Figure 8:**
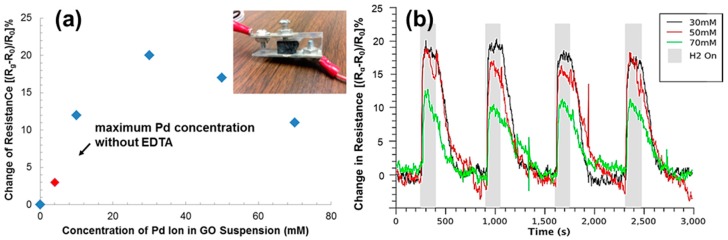
(**a**) The change of resistance of GA/Pd nanoparticle composites with different Pd loadings upon exposure to 1000 ppm hydrogen gas. (**b**) The change of resistance of GA/Pd nanoparticle composites upon hydrogen gas on/off cycles.

The chemical states of the metal nanoparticles loaded onto the aerogels, before and after hydrogen reduction, were analyzed via XPS, and the data are presented in Figure 7. The XPS spectra of palladium, platinum, nickel, and tin in aerogels before hydrogen reduction are presented in Figure 7a,c,e,g, respectively. The XPS spectra of palladium, platinum, nickel, and tin in aerogels after hydrogen reduction are presented in Figure 7b,d,f,h, respectively. The deconvoluted XPS spectra in Figure 7a,b show 3d peaks of palladium before and after hydrogen reduction. In Figure 7a, the deconvoluted XPS peaks are recognized as Pd^2+^ 3d_5/2_ peak (338.55 eV) of palladium (II) chloride (PdCl_2_), Pd^2+^ 3d_3/2_ peak (344.71 eV) of PdCl_2_, and Pd^2+^ 3d_5/2_ peak (337.19 eV) of palladium (II) oxide (PdO) [48]. The presence of a small portion of PdO is most likely due to the hydrolysis of PdCl_2_. In Figure 7b, the major deconvoluted XPS peaks are recognized as Pd° 3d_5/2_ peak (335.20 eV) and Pd° 3d_3/2_ peak (340.46 eV) of metallic palladium. These two major XPS peaks observed in palladium loaded aerogels after hydrogen reduction verifies the effective formation of metallic palladium during reduction [49]. Additionally, minimal deconvoluted XPS peaks corresponding to PdO are observed in Figure 7b, namely Pd^2+^ 3d_5/2_ peak (337.20 eV) and Pd^2+^ 3d_3/2_ peak (342.50 eV) of PdO [50]. The PdO in the aerogels after hydrogen reduction is likely from the surface oxides. The deconvoluted XPS spectra in Figure 7c,d show 4f peaks of platinum before and after hydrogen reduction. The deconvoluted XPS peaks in Figure 7c are recognized as Pt^4+^ 4f_7/2_ peak (74.56 eV), Pt^4+^ 4f_5/2_ peak (77.87 eV), Pt^2+^ 4f_7/2_ peak (71.99 eV), and Pt^2+^ 4f_5/2_ peak (75.50 eV), respectively [51,52,53]. The existence of peaks for Pt^2+^ is likely due to the reduction of Pt^4+^ during the step of mixing Pt^4+^ salts with EDTA [51]. Two major deconvoluted XPS peaks in Figure 7d are recognized as Pt° 4f_7/2_ peak (70.81 eV) and Pt° 4f_5/2_ peak (74.22 eV), respectively [52,53,54]. This result verifies the effective reduction to form metallic platinum. Besides, minimal deconvoluted XPS peaks corresponding to platinum (II) oxide (PtO) are observed in Figure 8d, namely Pt^2+^ 4f_7/2_ peak (71.98 eV) and Pt^2+^ 4f_5/2_ peak (75.45 eV). Similar to the case of palladium loaded aerogels, the PtO in the aerogels after hydrogen reduction is also likely from the surface oxides. The deconvoluted XPS spectra in Figure 7e,f show 2p peaks of nickel before and after hydrogen reduction. In Figure 7e, the single major peak is recognized as Ni^2+^ 2p_3/2_ peak (860.20 eV) [55,56]. In Figure 7f, the deconvoluted XPS peaks are recognized as Ni° 2p_3/2_ peak (858.63 eV), Ni^2+^ 2p_3/2_ peak (860.21 eV), and Ni^3+^ 2p_3/2_ peak (861.40 eV), respectively [57]. The Ni° 2p_3/2_ peak (858.63 eV) is the major deconvoluted XPS peak, and this result verifies the effective reduction to form metallic nickel. Besides, the existence of Ni^2+^ and Ni^3+^ after hydrogen reduction is probably attributed to the formation of nickel (II) oxide (NiO) and nickel (III) oxide (Ni_2_O_3_) on the surface of aerogels [51]. The deconvoluted XPS spectra in Figure 7g,h show 3d peaks of tin before and after hydrogen reduction. In Figure 7g, the deconvoluted XPS peaks are recognized as Sn^4+^ 3d_5/2_ peak (487.09 eV) and Sn° 3d_5/2_ peak (484.95 eV), respectively [58,59,60,61]. The presence of a small amount of metallic tin is likely due to the reduction by EDTA while mixing tin salts with EDTA. In Figure 7h, the single major peak is recognized as Sn° 3d_5/2_ peak (484.96 eV) [61], and this result verifies the effective reduction to form metallic tin. 

Overall, the effective reduction of metal salts during a hydrogen reduction process was confirmed by the comparison of the deconvoluted XPS peaks before and after reduction. The appearance of dominant XPS peaks, which correspond to the metals at their metallic state, indicate the highly effective reduction of the metal salts in this study. Additionally, only a small amount of metal oxides exist in the aerogels loaded with palladium, platinum, and nickel after hydrogen reduction, and the residual metal oxides are probably attributed to oxides formed on the surface after hydrogen reduction. Furthermore, no XPS peaks regarding tin oxides were seen while applying curve fitting, thus the reduction to form metallic tin nanoparticles is more effective than that of the other three kinds of metals.

The reduction of carbon in aerogels during hydrogen reduction was also confirmed by the XPS spectra of C 1 s. The XPS images of carbon in aerogels loaded with four different kinds of metals are shown in Figure S1. The obvious shift of XPS peaks to the lower binding area proves the reduction of GO during the reduction. For aerogels loaded with palladium, platinum, and nickel, the presence of major deconvoluted peaks at the binding energy of 284.6 eV, which is the signature peak of graphitic C–C bonds [25,37,62,63], indicates the effective reduction of carbon in hydrogen atmosphere at the temperature of 900 °C. Besides, the dramatic decrease of the relative intensity for the XPS peaks which correspond to the oxidized carbons, namely peaks of hydroxyl groups with binding energy around 286.5 eV, peaks of carbonyl C=O double bond with binding energy of around 288 eV, and peaks from O–C=O ester functional groups with binding energy around 289 eV [25,37,62,63], further verifies the effective reduction of carbonyl and ester groups. For tin loaded aerogels, the appearance of the deconvoluted XPS peak at the binding energy of 284.6 eV—which is the characteristic peak of graphitic carbon—proves the effective reduction of carbon in hydrogen atmosphere. Because a much lower reduction temperature of 400 °C was used for tin loaded aerogels, a much smaller fraction of graphitic carbon was formed. Additionally, the decrease of the relative intensity of the ester groups verifies the reduction of this functional group in aerogels. At last, deconvoluted peaks with the binding energies of 290.1 eV to 290.8 eV, which exist in all aerogel samples as prepared and after hydrogen reduction, are the characteristic π–π shakeup transition peaks in aromatic carbon compounds [63,64].

Pd nanoparticles have been deposited on GO surfaces for hydrogen sensing because they have high affinity to hydrogen. Such affinity causes the electron density of the metal particle to be reduced, thereby increasing the work function of the graphene-metal-gas system, leading to a change of resistance [47,65]. The sensitivity of the sensors depends on the Pd nanoparticle/GO interfacial area and the response and recovery time is affected by the gas flow efficiency. The Pd nanoparticles on GAs offer a large contact area between Pd nanoparticles and graphene and efficient gas flow due to the porous structures. In our study, different amount of Pd nanoparticles were loaded onto rGO aerogels (rGOAs) by adding different amount of PdCl_2_/EDTA solutions to GO suspensions. In addition, PdCl_2_ without EDTA was added to GO suspensions as a control. While the concentration of PdCl_2_ can be as high as 70 mM with EDTA before GO is precipitated, more than 4 mM pure PdCl_2_ would precipitate GO. The produced rGOA composites were put between two electrodes and subjected to hydrogen sensing testing as shown in the Figure 8a inset. The resistance change of each rGOA/Pd nanoparticle composite upon the exposure to 1000 ppm hydrogen gas is shown inFigure 8a. First, heavy loading of Pd nanoparticles on rGOA using EDTA have a much higher response to hydrogen gas than small loading of Pd nanoparticles using pure PdCl_2_, suggesting that increased Pd nanoparticles on rGOA can increase sensitivity. Second, the sensor response increases with Pd nanoparticle loading reach a peak at 30 mM Pd precursor concentration, and then decreases. With increasing Pd loading, the amount of nanoparticles increases, the resulting increased Pd nanoparticle surface area leads to an increase in sensitivity. However, the Pd nanoparticle size will increase with further increased Pd loading (Figure S2), which causes the reduction of Pd nanoparticle surface area. The surface area of compoiste rGOA fabricated from 30 mM and 70 mM was 328 m^2^/g and 249 m^2^/g, respectively. Figure 8b shows the change of resistance of rGOA/Pd nanoparticle composites versus time over four on-off cycles of 1000 ppm hydrogen gas. The performance comparison of our hydrogen sensor to the reported hydrogen sensors using Pd nanoparticles is listed in Table 1. It clearly shows a fast response of hydrogen gas (25 s) and recovery (175 s) (Figure S3). We believe that the improved response and recovery rate of our hydrogen senors is attributed to the increased Pd loading and enhanced gas flow efficiency in rGOAs. Larger amount of Pd nanoparticles provide more binding sites for hydrogen gas and porous structure of rGOA facilitates the accessibility of hydrogen gas to Pd nanoparticles. 

## 4. Conclusions

In this paper, rGO aerogels loaded with four kinds of metallic nanoparticles were successfully prepared with excellent directionality and decent hieratical structures. The loading of 59, 67, 39, and 46 wt % of metallic palladium, platinum, nickel, and tin in rGO/metal aerogels was achieved after hydrogen reduction at elevated temperatures. It is believed that other kinds of metallic nanoparticles can also be loaded onto rGO aerogels with similar procedures. A hydrogel gas sensor was produced using Pd nanoparticle loaded rGO aerogels. The large surface area and porous structure of rGO aerogels offer high sensitivity and fast response/recovery time of the sensor. The metal nanoparticle decorated aerogels which have open-pore structure and a large surface area are promising platforms for numerous applications, including energy storage, gas sensing, and catalysts of vapor phase reactions.

## Figures and Tables

**Table 1:** Comparison of hydrogen gas sensing performance.

Material	Method	Response Time	Recovery Time	Detection Range	Reference
Pd nanoparticles on CNTs	chemical reduction	18 min. (300 ppm) and 7 min (3000 ppm)	20 min (100 ppm) and 55 min (1000 ppm)	100–1000 ppm	[66]
Pd and C60	physical vapor deposition	500 s	500 s	1%	[67]
Pd nanocubes on CNTs	chemical reduction	15 min	5 min.	10 ppm–1%	[68]
Pd nanoparticles on graphene	galvanic displacement reaction	2 min	5 min	25 ppm–2%	[69]
Pd nanoparticles on graphene nanoribbon films	chemical reduction	750 s	tens of min	100 ppm–1%	[70]
Pd nanoparticles on graphene nanoribbon films	e-beam evaporation	15 min	10 min	40–8000 ppm	[71]
Pd nanoparticles on graphene aerogels	chemical reduction	25 s	175 s	100–1000 ppm	current work

## Supplementary Materials

The following are available online at www.mdpi.com/2072-666X/8/2/47/s1.
