# Supplementary material for: Fabrication of Graphene Aerogels with Heavily Loaded Metallic Nanoparticles
Source: Micromachines (Basel). 2017 Feb 7;8(2):47. doi: 10.3390/mi8020047 (PMC6190163; doi:10.3390/mi8020047)
Supplement: Supplementary file 1 [file micromachines-08-00047-s001.pdf]

Supplementary Materials: Fabrication of Graphene Aerogels with Heavily Loaded Metallic Nanoparticles

Chen Shen, Elizabeth Barrios, Matthew McInnis, Joseph Zuyus and Lei Zhai

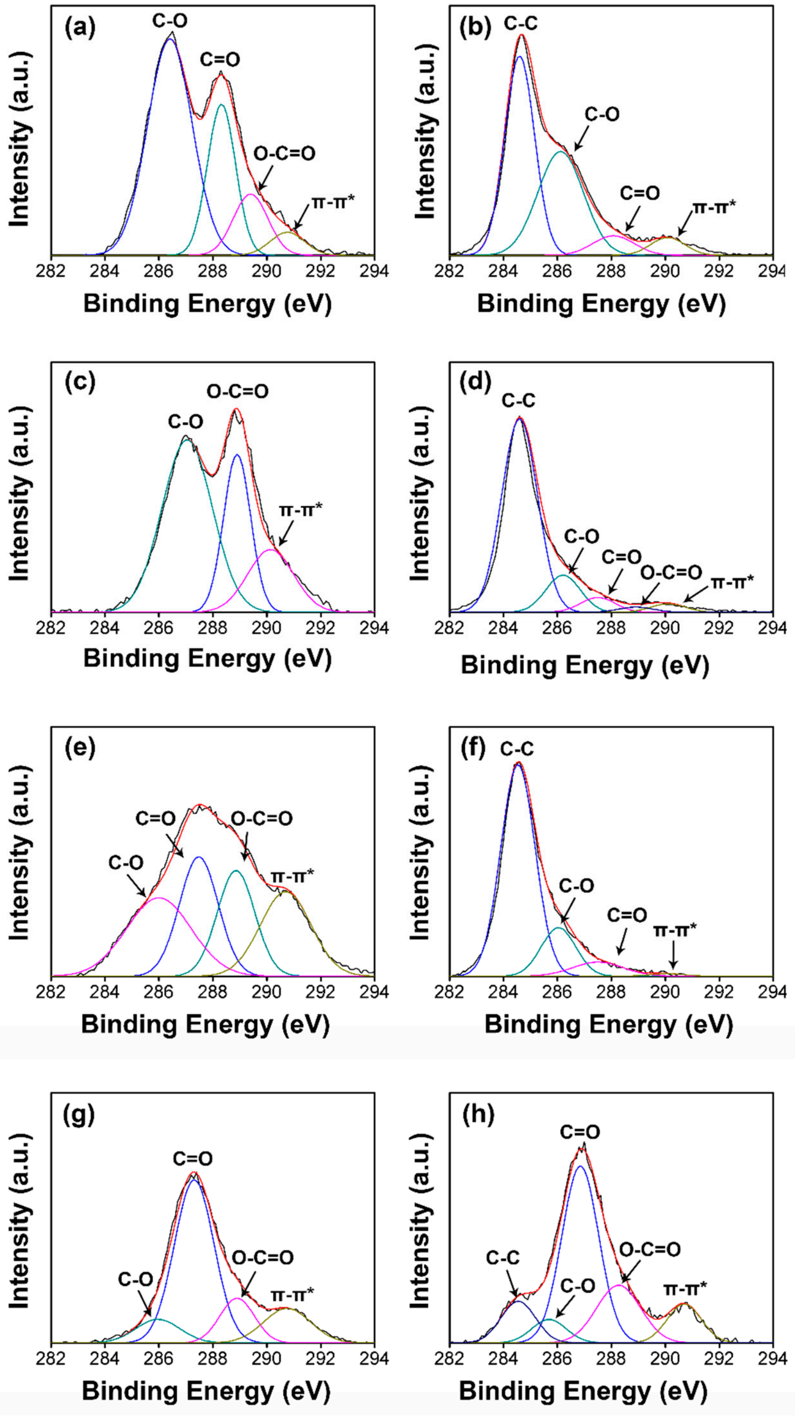

Figure S1. Deconvoluted XPS spectra of the carbon in the aerogels. C in Pd loaded aerogels (**a**) as prepared and (**b**) after reduction, C in Pt loaded aerogels (**c**) as prepared and (**d**) after reduction, C in Ni loaded aerogels (**e**) as prepared and (**f**) after reduction, C in Sn loaded aerogels (**g**) as prepared and (**h**) after reduction.

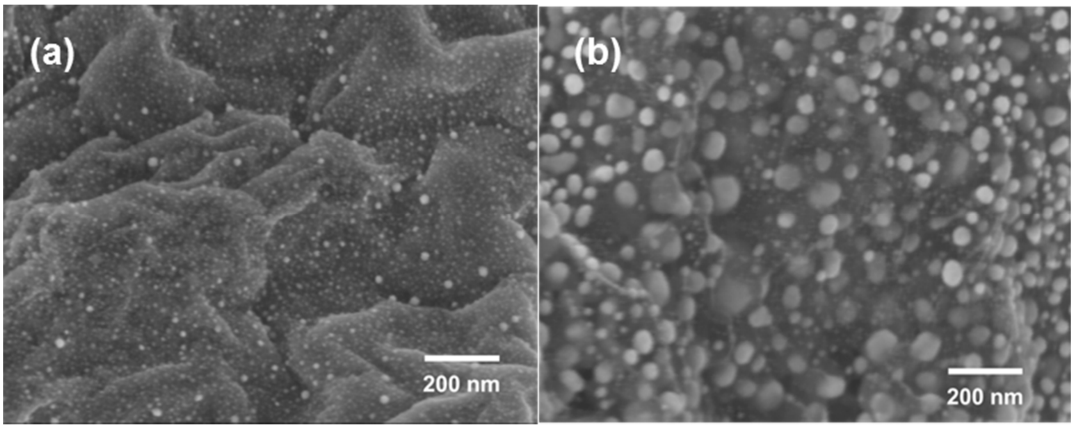

Figure S2. SEM images of Pd nanoparticles on GA surfaces made from (**a**) 30 mM and (**b**) 70 mM Pd ion in GO suspensions.

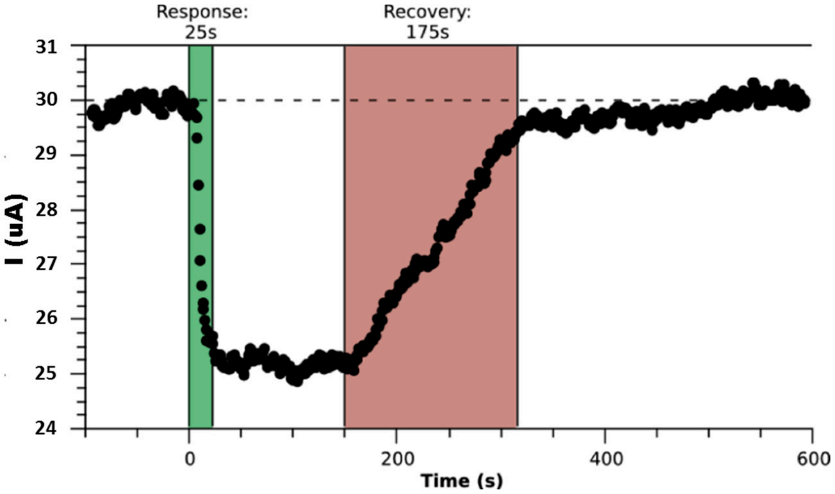

Figure S3. The change of the current of a hydrogen sensor when exposed to 1000 ppm hydrogen gas. The responding time and recovery time are highlight in green and brown, respectively.
